# Transient Psychosis in Chemsex: Mephedrone and Methamphetamine Use in a Clinical Case and Observational Study

**DOI:** 10.1192/j.eurpsy.2026.10867

**Published:** 2026-07-31

**Authors:** M. Ligero Argudo, I. M. Peso Navarro, C. García Cerdán, A. I. Mitadiel Velasco, C. Marín Lorenzo, N. Casado Espada, J. I. de la Iglesia Larrad, C. Munaiz Cossío, R. K. González Bolaños, C. Payo Rodríguez

**Affiliations:** 1 Psychiatry; 2 Psychology, Hospital Clínico de Salamanca, Salamanca, Spain

## Abstract

**Introduction:**

Chemsex, defined as the use of drugs to enhance sexual experiences, has gained attention due to its physical and psychological risks. Mephedrone and methamphetamine are among the most commonly used substances and have been associated with neuropsychiatric alterations, including psychotic symptoms. Understanding this relationship is essential for preventive and therapeutic strategies.

**Objectives:**

To analyze the prevalence of mephedrone and methamphetamine use among individuals engaging in chemsex, their association with psychotic symptoms, and to illustrate these effects through a clinical case.

**Methods:**

We present a 59-year-old male with well-controlled HIV (undetectable viral load) and hepatitis C. He initiated intravenous mephedrone and smoked methamphetamine in the context of chemsex. During episodes of substance use, he experienced acute psychotic symptoms including paranoia, delusional ideation, and perceptual disturbances (visual and auditory hallucinations). No prior psychiatric history was reported. Symptoms were temporally linked to substance use and resolved during periods of abstinence. The patient received supportive care and psychoeducation regarding the neuropsychiatric risks of chemsex.

**Results:**

Supportive interventions, including counseling and psychoeducation on substance risks, were provided. Over a follow-up period of three months with abstinence from chemsex drugs, the patient remained free of psychotic symptoms, suggesting a strong temporal relationship between substance use and acute psychosis.

**Conclusions:**

Mephedrone and methamphetamine use in chemsex is associated with transient psychotic symptoms. Frequency, substance combination, and intravenous administration are key risk factors. The case report highlights the clinical presentation of psychosis linked to chemsex, emphasizing the need for targeted mental health interventions, harm reduction programs, and education on neuropsychiatric risks.

**Disclosure of Interest:**

None Declared

